# A Comparative Study to Assess the Awareness of Palliative Care Between Urban and Rural Areas of Ernakulum District, Kerala, India

**DOI:** 10.4103/0973-1075.58457

**Published:** 2009

**Authors:** Nitin Joseph, Jayarama S, Shashidhar Kotian

**Affiliations:** Department of Community Medicine, Kasturba Medical College, Mangalore, India

**Keywords:** Awareness, Comparative study, Palliative care, Rural areas, Urban areas

## Abstract

**Aim:**

To assess the knowledge and attitude toward palliative care among people residing in urban and rural areas.

**Materials and Methods:**

This cross-sectional study was conducted among 185 urban and 165 rural households. Senior-most member of the household present was interviewed using a questionnaire. Only those people who have heard about palliative care were included in the study.

**Results:**

Out of 350 people, 47 (13.4%) have heard about palliative care. Of these 38 (20.5%) belonged to urban and 9 (5.4%) belonged to rural areas (*P* < 0.0001).Twenty-nine (15.7%) participants in urban and 7 (4.2%) in rural areas had some knowledge about palliative care (*P* = 0.0002). Source of information for 25 (53.2%) participants was newspapers followed by television 17 (36.2%). Thirty-three (86.8%) participants in urban and 7 (77.8%) in rural areas felt that palliative care helps in improving quality of life. Twenty (52.6%) participants in urban and 4 (44.4%) participants in rural areas felt that palliative care can be better provided at homes than hospitals. Thirty (78.9%) urban participants felt that bad news about the patient's condition needs to be told to the patient first and then to their family members. In case of rural participants majority 7 (77.8%) said vice versa (*P* = 0.0039).

**Conclusion:**

Overall awareness of palliative care was poor. This calls for large-scale awareness campaigns. As home-based palliative care was preferred by many, home visits by care providers and training of family members of patient's needing palliative care needs to be practiced widely.

## INTRODUCTION

Palliative care is an approach that improves the quality of life of patients and their families facing life threatening illnesses.[1] It is derived from the Latin word ‘palliare’ means ‘to cloak’.[2] The approach involves reducing the severity of disease symptoms so as to offer relief in suffering, psychosocial care to handle depression, and spiritual care for patients to take life positively.[1]

Palliative treatment may also be used to alleviate the side effects of curative treatment such as relieving the nausea associated with chemotherapy in cancer patients.[2]

The care aims to maintain dignity of the patient even in death and to sustain and rehabilitate the individual's family. For example, bereavement counseling to the patient's family should he/she die.[3]

Palliative care is increasingly used with diseases like cancer, chronic progressive pulmonary disorders, renal diseases, chronic heart failure, and progressive neurological conditions.[2]

Palliative care began in hospice movement. These hospices were originally places of rest for travelers in the fourth century. The modern hospice movement was founded by Dame Cicely Saunders of England in 1967. The first hospital-based palliative care program began in United States in late 1980s. In most countries, palliative care is provided by an interdisciplinary team consisting of physicians, registered nurses, nursing assistants, social workers, hospice chaplains, physiotherapists, occupational therapists, complementary therapists, volunteers, and most importantly family members.[2]

Palliative care is a new concept in our society. This research attempts to find out and compare knowledge and attitude toward palliative care and its contributing factors among people living in urban and rural areas of Ernakulum District in Kerala.

## MATERIALS AND METHODS

The present study was undertaken in Ernakulum district, which comes in the central part of Kerala state. The literacy status in this district is 93.2%.[4] Pain and palliative care is gaining momentum in this district with 15 pain and palliative care units functioning till the end of 2008. The movement is well supported by the district panchayat, grama panchayats, and other local bodies and NGOs. In Ernakulam district, however, palliative care is mainly hospital-based while in Kozhikode and Malappuram districts of Kerala, where the movement has deep roots, the focus in on house call.[5] Hence it is still a long way for this district to have a community-based palliative care service.

The Kaloor town and Njarakkal village of this district were chosen as study areas in order to get an urban and rural background in this comparative study. Kaloor town has a population of 13,082 with 2903 households. Njarakkal village has a population of 24,166 with 5276 households.[4] Approximately 5% of households were chosen by systematic random sampling method. In Njarrakal, due to difficult topography of the region only 165 (3.1%) of houses could be visited in 1 week. This was compensated in Kaloor town; 185 (6.4%) houses were covered during the study period.

It was a cross-sectional study wherein the senior-most member of the household present at the time of interview was interviewed using a predesigned and pretested questionnaire.

Informed consent was taken from them for their willingness to participate in the study.

Those people who had heard about palliative care formed the inclusion criteria, referred to as participants while those who have not heard about palliative care formed the exclusion criteria.

Data analysis was done using SPSS ver 15.0, statistical test Chi square was done and *P* < 0.05 were taken as statistically significant.

## RESULTS

Out of the 350 people visited, 185 belonged to Kaloor town and 165 belonged to Njarakkal village. All of them were literates with a minimum education of at least fifth standard but only 47 (13.4%) of them have heard about palliative care. Out of these, 38 (20.5%) belonged to urban and 9 (5.4%) to rural areas. This difference was found to be statistically significant (χ^2^ = 17.074, *P* < 0.0001).

When the participants were enquired about their level of knowledge, 29 (15.7%) of urban and 7 (4.2%) of rural participants said that they had some knowledge about palliative care. This difference was also found to be statistically significant [Table 1].

**Table 1:** Level of knowledge about palliative care

Level of knowledge	Urban population no. (%)	Rural population no. (%)	Total no. (%)
Nil	147 (79.5)	156 (94.5)	303 (86.7)
Poor	9 (4.8)	2 (1.3)	11 (3.1)
Moderate	29 (15.7)	7 (4.2)	36 (10.2)
Total	185 (100.0)	165 (100.0)	350 (100.0)

χ^2^ = 17.079; *P* = 0.0002

Most of the participants 25 (53.2%) came to know about palliative care through newspapers followed by television 17 (36.2%). In rural areas, one-third of the participants got this information from health workers [Table 2].

**Table 2:** Source of information about palliative care

Source of information	Urban participants (*n* = 38) no. (%)	Rural participants (*n* = 9) no. (%)	Total (*n* = 47) no. (%)
Newspaper	21 (55.3)	4 (44.4)	25 (53.2)
TV	14 (36.8)	3 (33.3)	17 (36.2)
Health workers	5 (13.2)	3 (33.3)	8 (17.0)
Friends	7 (18.4)	0 (0.0)	7 (14.9)
Internet	0 (0.0)	1 (11.1)	1 (2.1)
Others*	13 (34.2)	2 (22.2)	15 (31.9)

* Workshops, magazines, church, posters and books

All the participants were aware that palliative care was meant for chronic diseases. Of this most of them 34 (72.3%) said that it was meant for patients with mental illnesses (including stroke) followed by cancer 33 (70.2%) and AIDS 23 (48.9%) [Table 3].

**Table 3:** Knowledge of diseases requiring palliative care

Diseases	Urban participants no. (%)	Rural participants no. (%)	Total no. (%)
CNS disorders	28 (73.7)	6 (66.7)	34 (72.3)
Cancer	26 (68.4)	7 (77.8)	33 (70.2)
AIDS	17 (44.7)	6 (66.7)	23 (48.9)
Cardiovascular ailments	10 (26.3)	4 (44.4)	14 (29.8)
Respiratory ailments	11 (28.9)	4 (44.4)	15 (31.9)

Most of the participants in both urban and rural areas 43 (91.5%) were aware that palliative care helps in improving the quality of life of the patient, while 7 (14.9%) felt that it prolongs the period of survival [Table 4].

**Table 4:** Benefits derived out of palliative care

Benefits	Urban participants no. (%)	Rural participants no. (%)	Total no. (%)
Prolongs the period of survival	3 (7.9)	1 (11.1)	4 (8.5)
Improves the quality of life	33 (86.8)	7 (77.8)	40 (85.1)
Both	2 (5.3)	1 (11.1)	3 (6.4)
Total	38 (100.0)	9 (100.0)	47 (100.0)

X^2^ = 0.549; *P* = 0.7601

Most of the urban participants 16 (42.1%) wanted information about palliative care to be made more generally available while most of the rural participants 4 (44.4%) wanted this information only at the time of diagnosis of an incurable disease. However, these differences in attitude were not found to be statistically significant [Table 5].

**Table 5:** Time for requirement of palliative care in case of incurable illness

	Urban participants no. (%)	Rural participants no. (%)	Total no. (%)
At diagnosis	15 (39.5)	4 (44.4)	19 (40.4)
Terminally	7 (18.4)	2 (22.2)	9 (19.1)
More generally available	16 (42.1)	2 (22.2)	18 (38.3)
Never	0 (0.0)	1 (11.1)	1 (2.2)
Total	38 (100.0)	9 (100.0)	47 (100.0)

X^2^ = 1.556; *P* = 0.459

Most of the participants 24 (51.1%) felt that palliative care can be best administered in the home environment [Table 6].

**Table 6:** Opinion about best place for providing palliative care

Place	Urban participants no. (%)	Rural participants no. (%)	Total no. (%)
At home	20 (52.6)	4 (44.4)	24 (51.1)
At hospital	16 (42.1)	4 (44.4)	20 (42.6)
Both	2 (5.3)	1 (11.1)	3 (6.4)
Total	38 (100.0)	9 (100.0)	47 (100.0)

X^2^ = 0.495; *P* = 0.781

All the rural participants and 35 (92.1%) of urban participants felt that the treating doctor should honestly tell the patient about his/her condition. But when it came to who should be first informed about the bad news, the attitude differed between urban and rural participants. Majority of rural participants 7 (77.8%) said ‘to family first’, while majority of urban participants 30 (78.9%) said ‘to patient first’. This difference in attitude was found to be statistically significant (χ^2^ = 8.322, *P* = 0.0039).

When asked about their interest to know further about palliative care, 31 (81.6%) of urban participants and all the rural participants expressed their willingness to learn more.

## DISCUSSION

Concept of palliative care is relatively new in India, but the requirement is beyond our imagination. With improved standards of living and adoption of western habits such as tobacco use and alcoholism, the numbers of cancer cases are increasing in India.[6] It is estimated that around 2.5 million people suffer from cancer at any given time in India. Almost 80% of patients reach hospitals in advanced stages of the disease.[7] This has led to a mounting need for palliative care. Lately the explosion in HIV/AIDS cases in India has made the requirement for palliative forms of treatment even more acute.[6]

In our study, only 13.4% of people had heard about palliative care. This finding was similar to a study done by Kolchakova[8] in which 15% of people heard about palliative care. In another study done by Canadian Hospice Palliative Care Association,[9] as much as 53% of people have heard about palliative care. Of them, 30% of people had some knowledge about palliative care. This observation was much higher than the findings in our study as only 10.3% of population had some knowledge about palliative care. Canada being a developed country would have been facing the problems of noncommunicable diseases since long. This would have resulted in their population being much more acquainted with palliative care services.

Source of information about palliative care for majority of urban and rural participants was newspapers and television. This makes these media as popular means to propagate awareness in community about palliative care. Health workers being responsible for giving information to a good number of rural participants gives importance to the vital role played by them in such difficult-to-access areas.

In Kolchakova[8] study, 98% of participants knew that palliative care was meant for patients with incurable diseases. They also knew that palliative care services target the family members of the patients. In our study also, 72.3%, 70.2%, and 48.9% of respondents told that palliative care is meant for diseases like CNS disorders, cancer, and HIV/AIDS, respectively.

In this study, 40.4% participants felt that the best time to give information about palliative care was at the time of diagnosis of disease condition, while 38.3% felt that it should be made more generally available. In the study done by Wallace,[10] majority of participants, ie., 64.9%, felt that the information should be made more generally available.

Most of our participants, ie. 51.1%, felt that palliative care can be given best at homes than at hospitals. In a study done by Uwimana,[11] 62% of PLWHA preferred home-based palliative care over hospital-based care services. Another observation made by him was that over half of healthcare professionals were not trained in palliative care.

Even though the awareness about palliative care is less among participants in our study, the enthusiasm expressed by them in learning and involving in palliative care services is commendable. Total 85.1% of our participants expressed interest to learn more and involve themselves in palliative care activities. This finding was similar to findings of Kolchakova[8] where 67% of participants expressed willingness to be involved as volunteers. Every effort should thus be made to empower the family members of the patient and involve them in decision making with regard to treatment.[12]

All these are a waking call for palliative care professionals to train grass root level workers chosen from the community, patient's relatives, and other volunteers in palliative care so that they can actively render their services for home-based palliative care activities.

Most participants (93.6%) in our study felt that the treating doctor should give an honest opinion to the patient about his/her condition. This was much higher than the findings of Heyland[13] where only 44.1% of the participants felt the same.

Also 78.9% of our urban participants felt that bad news about patient's condition should be told to the patient first, while 77.8% of rural participants felt that it should be told first to family members. This reveals the cultural difference between people living in urban and rural areas. In rural areas, the family values and support are more prioritized over individual importances.

Obstacles identified in Uwimana[11] study by providers of palliative care were shortage of equipments, prevailing culture, stigma and inadequate policy on palliative care, as well as opiod drugs. The above study also identified differences in palliative care needs between urban and rural PLWHAs. This difference occurred due to differences in levels of poverty, traditional, cultural, and religious values and belief systems based on area of residence. Such findings also hold good in the state of Kerala in spite of its good literacy record. This has been supported by our study results. This makes addressing these requirements of palliative care important while rendering our services in various parts of Kerala in future.

## CONCLUSION

Utility of health services is still inadequate in most parts of India. Lack of resources, illiteracy, poverty, lack of awareness about the types of available healthcare facilities among people make developing palliative-care services a major challenge in India.[14] The situation is more acute in rural areas of India where about two-third of population resides.

This was obvious in the study results which showed the level of knowledge significantly high among urban than rural participants. However, the overall awareness of palliative care was poor among all participants in spite of a good literacy status in the study area. This means our health educational system needs to be updated regularly. As most of the participants in our study wanted the information about palliative care to be made more generally available, the popular sources of information like newspapers and television should be used to disseminate information on palliative care on a large scale. This will also improve the utility of palliative care services provided by us among the people. The attitudinal variation regarding to whom the bad news of the patient's condition needs to be told first, ie. to the family or to the patient also differed in urban and rural areas. This difference in expectation toward palliative care means that the type of service should be tailor-made based on area of our activity.

Majority of participants felt that palliative care can be best given at homes rather than hospitals. The aim should, therefore, be to train a good number of doctors, nurses, community workers, and volunteers in palliative care based on our requirements. Training should also target communication skills and proper manner of breaking bad news to patients and their family members as well.[12]
